# HMGB1 induces hepcidin upregulation in astrocytes and causes an acute iron surge and subsequent ferroptosis in the postischemic brain

**DOI:** 10.1038/s12276-023-01111-z

**Published:** 2023-11-01

**Authors:** Dashdulam Davaanyam, Hahnbi Lee, Song-I Seol, Sang-A Oh, Seung-Woo Kim, Ja-Kyeong Lee

**Affiliations:** 1 Department of Anatomy, Inha University School of Medicine, Incheon, 22212 Korea; 2 Department of Biomedical Sciences, Inha University School of Medicine, Inchon, 22212 Korea

**Keywords:** Stroke, Acute inflammation

## Abstract

Dysregulation of brain iron levels causes functional disturbances and damages neurons. Hepcidin (a peptide hormone) plays a principal role in regulating intracellular iron levels by modulating ferroportin (FPN, the only known iron exporter) through triggering its internalization and lysosomal degradation. We observed a significant and rapid iron surge in the cortices of ischemic hemispheres at 3 h after cerebral ischemia (middle cerebral artery occlusion, MCAO) that was maintained until 4 d post-MCAO. We showed upregulation of hepcidin expression in the brain as early as 3 h post-MCAO, mainly in astrocytes, and significant hepcidin accumulation in serum from 6 h post-MCAO, and these inductions were maintained for 1 day and 7 days, respectively. High mobility group box 1 (HMGB1), a prototypic danger-associated molecular pattern, accumulates markedly after transient MCAO and plays critical roles in damage aggravation via its proinflammatory effects. Here, we demonstrated that treatment with recombinant HMGB1 stimulated astrocytes to induce hepcidin expression in a TLR4- and CXCR4-dependent manner. Furthermore, hepcidin-mediated intracellular iron accumulation in neurons was confirmed by an experiment using N-methyl-D-aspartate (NMDA)-conditioned medium-treated primary astrocytes and fresh primary cortical neurons treated with hepcidin-containing astrocyte-conditioned medium. Moreover, HMGB1-mediated local hepcidin upregulation and subsequent local iron surge were found to cause ferroptosis in the postischemic brain, which was suppressed by the functional blocking of HMGB1 using intranasally administered HMGB1 A box or anti-HMGB1 antibody. These findings show that HMGB1 serves as a ferroptosis inducer by upregulating hepcidin in astrocytes and thus aggravates acute damage in the postischemic brain.

## Brain iron dysregulation: a culprit in neuron damage

Researchers have discovered that the High Mobility Group Box 1 (HMGB1) protein plays a key role in the regulation of iron levels in the brain, which can contribute to the severity of stroke damage. The study found that blocking HMGB1 function with an anti-HMGB1 antibody or HMGB1 A box suppressed ferroptosis, a type of cell death, in the post-ischemic brain. This finding suggests that targeting HMGB1 could potentially mitigate ischemic brain damage by reducing iron accumulation and subsequent ferroptosis in neurons. The study provides new insights into the molecular mechanisms underlying iron regulation in the brain and highlights potential therapeutic targets for stroke treatment.

## Introduction

Iron participates in many vital functions in the brain, including cellular respiration, metabolism, neurotransmitter production, and myelin synthesis^1,2^. However, iron is highly toxic in excess due to the Fenton reaction, and the dysregulation of iron levels in the brain causes functional disturbances and damages neurons^1–3^. An increase in intracellular iron levels has been detected in various pathological conditions of the CNS, including Alzheimer’s disease^4,5^, Parkinson’s disease^6,7^, and Huntington’s disease^8^. Neuronal damage induced by dysregulation of cellular iron homeostasis has also been reported after stroke^9–11^, and elevated iron levels have also been observed in stroke patients^12,13^. Changes in intracellular iron levels may be due to a surge in iron release from damaged cells or an influx from blood due to BBB (blood brain barrier) impairment^3^. In addition, dysregulation of molecules involved in maintaining cellular iron homeostasis can cause intracellular iron accumulation by enhancing iron uptake or suppressing its efflux^14,15^.

Among the cellular and molecular components involved in the modulation of intracellular iron levels, hepcidin (a peptide hormone) plays a principal role in regulating iron export by triggering the internalization and lysosomal degradation of ferroportin (FPN), the only known iron exporter^16^. Hepcidin is synthesized mainly by hepatocytes in response to iron signals, inflammation, hypoxia, and endoplasmic reticulum stress^17^. However, its expression has been reported at low levels in other tissues, including the retina^18^, heart^19^, kidneys^20^, and brain^21,22^. Serum hepcidin levels are elevated in acute neuropathological conditions such as acute ischemic stroke or intracerebral hemorrhage^10,12,13,23,24^. Furthermore, hepcidin has been reported to have deteriorating effects in iron-loaded ischemia models, in which hepcidin administration exacerbated and hepcidin knockdown ameliorated brain damage^25^. In addition, aberrant hepcidin expression has been reported in Alzheimer’s disease^26,27^, Parkinson’s disease^28,29^, multiple sclerosis^30^, and brain cancer^31^.

In the ischemic brain, the inflammatory response aggravates brain damage^32^. IL-6 (a key proinflammatory cytokine) has been shown to upregulate hepcidin in astrocytes and thus to downregulate FPN levels in the brain^33–35^, which supports the critical role of inflammation in inducing local hepcidin expression in the brain. We previously reported that HMGB1 (high mobility group box 1), a proinflammatory, prototypic danger-associated molecular pattern molecule, accumulates in the brain parenchyma, cerebrospinal fluid (CSF), and serum after transient middle cerebral artery occlusion (MCAO) and aggravates brain damage^36–38^. HMGB1 has two redox state-dependent forms, a disulfide-type HMGB1 (dsHMGB1) and a reduced-type HMGB1 (reHMGB1)^39,40^. dsHMGB1 is elevated in the postischemic brain and exerts its proinflammatory function by binding to TLR4 (Toll-like receptor 4)^41^, which interestingly has been reported to upregulate hepcidin expression in lipopolysaccharide (LPS)-treated retinal pigment epithelium^18^ and hepatocytes^42^.

Although evidence shows that iron overload is a major source of oxidative stress in the ischemic brain and contributes to I/R-mediated damage^3,10,11,15^, the mechanism of local iron regulation during the acute to subacute periods in the ischemic brain remains unclear. In this study, we investigated whether HMGB1 enhances local hepcidin levels and the subsequent intracellular iron surge in the ischemic brain and sought to identify the cell types involved in hepcidin induction in the brain and intracellular iron accumulation. We also investigated the effects of early ferroptosis in an animal model of transient cerebral ischemia.

## Materials and methods

### Surgical procedure used for middle cerebral artery occlusion

Male Sprague–Dawley rats (7–8 weeks of age) were housed under diurnal lighting conditions and allowed food and tap water *ad libitum*. All animal studies were carried out in strict accordance with the recommendations of the Guide for the Care and Use of Laboratory Animals published by the National Institutes of Health (NIH, USA, 2013) and ARRIVE guidelines (http://www.nc3rs.org/ARRIVE (accessed on August 31, 2021). The animal protocol used was reviewed for ethicality and approved beforehand by the INHA University Institutional Animal Care and Use Committee (INHA-IACUC) (approval number INHA180105-531-2). MCAO was performed as previously described^43^. Briefly, 8-week-old male Sprague–Dawley rats (250–300 g) were anesthetized with 5% isoflurane in 30% oxygen/70% nitrous oxide and maintained using 0.5% isoflurane in the same gas mixture during surgery. The right middle carotid artery was occluded for 1 h by advancing a nylon suture (4-0; AILEE, Busan, Korea) with a heat-induced bulb at its tip (~0.3 mm in diameter) along the internal carotid artery 20–22 mm from its bifurcation with the external carotid artery, and this was followed by reperfusion for up to 7 d. During the procedure, the left femoral artery was cannulated to obtain blood samples, which were analyzed for pH, PaO_2_, PaCO_2_, and blood glucose (I-STAT; Sensor Devices, Waukesha, WI, USA) (Table 1). A laser Doppler flowmeter (Periflux System 5000; Perimed, Jarfalla, Sweden) was used to monitor regional cerebral blood flow (CBF) and relative CBF, and a thermoregulated heating pad and a heating lamp were used to maintain a rectal temperature of 37.0 ± 0.5 °C. The animals were randomly allocated to the following groups: sham (n = 40), MCAO 1 h (n = 17), MCAO 3 h (n = 17), MCAO 6 h (n = 35), MCAO 12 h (n = 25), MCAO 24 h (n = 20), MCAO 48 h (n = 17), MCAO 72 h (n = 14), MCAO 96 h (n = 11), MCAO 168 h (n = 4), MCAO + anti-HMGB1 antibody (n = 10), MCAO + HMGB1 A box (n = 10), MCAO + Deferoxamine (DFO) (n = 5), MCAO + Ferrostatin 1 (Fer1) (n = 5), MCAO + Hepcidin siRNA (n = 4), MCAO + Hepcidin siRNA + anti-HMGB1 antibody (n = 4), MCAO + IL-6 siRNA (n = 4), and MCAO + IL-6 siRNA + anti-HMGB1 antibody (n = 4). Animals in the sham group underwent an identical procedure without MCAO.

**Table 1:** Physiological parameters.

	Base	MCAO (12 h)
pH	7.45 ± 0.02	7.6 ± 0.13
pO_2_, mmHg	138.02 ± 3.85	144.9 ± 2.64
pCO_2_, mmHg	35.37 ± 0.84	35.05 ± 0.85
Glucose, mg/dL	108.86 ± 4.28	101.97 ± 1.58
Temperature °C	36.83 ± 0.2	36.96 ± 0.21

Values are means ± SDs (n = 3). One-way analysis of variance revealed no significant intergroup difference for any variance.

### Drug administration

Drugs were administered intranasally, as previously described by Kim et al.^41^. Briefly, rats were anesthetized with an intramuscular injection of a ketamine (3.75 mg/100 g body weight) and xylazine hydrochloride (0.5 mg/100 g per body weight) mixture. A nose drop containing HMGB1 A box (HM-012, 5 μg/kg; HMGbiotech, Milano, Italy), anti-HMGB1 antibody (200 µg/kg, Santa Cruz Biotechnology, Santa Cruz, CA, USA), ferrostatin-1 (Fer-1, 0.5 μg/kg, Sigma‒Aldrich, St. Louis, MO, USA), or deferoxamine (DFO, 100 mg/kg, Sigma‒Aldrich) dissolved in PBS (20 µL) was then carefully placed in each nostril of anesthetized animals (supine at a 90° angle) using a preautoclaved pipette tip (T-200-Y; Axygen, Union, CA, USA). The procedure was repeated until entire dosages were administered, with 2-min intervals between applications.

### Primary cortical neuron cultures

Experiments were conducted in accordance with the Guide for the Care and Use of Laboratory Animals published by the National Institutes of Health (NIH, USA, 2011). The animal protocol used in this study was reviewed and approved by the INHA-IACUC (approval number INHA 201103-734). All efforts were made to reduce the number of animals used and to minimize animal suffering. Briefly, mixed neocortical cells were prepared from embryonic Day 15.5 (E15.5) mouse cerebral cortices and cultured as previously described^36^. Cortical cells were dissociated using a glass Pasteur pipette and plated at a density of six hemispheres per well in 24-well poly-d-lysine (100 μg/mL) and laminin (100 μg/mL) (Thermo Fisher Scientific, Waltham, MA, USA)-coated plates (4 × 10^5^ cells per well). Cultures were maintained in minimum essential medium (MEM: Sigma‒Aldrich) containing fetal bovine serum (FBS, 5%), horse serum (5%), glucose (21 mM), and glutamine (2 mM) without antibiotics. On Day 7 in vitro (DIV7), when astrocytes had reached confluence underneath neurons, cytosine arabinofuranoside (ara-C, Sigma‒Aldrich) was added to a final concentration of 10 μM in MEM containing horse serum (10%) and glucose (21 mM). Cultures were maintained for 2 d to halt microglial growth. Media were changed on alternate days after DIV7, and FBS and glutamine were not supplemented from DIV7. Cultures were used on DIV12-14.

### Primary astrocyte cultures

Primary astrocyte cultures were prepared as previously described^44^. Briefly, the brains of newborn male Sprague–Dawley rats (<1 d old) were removed. Cortex tissue was dissociated, chopped, seeded into poly L-lysine-coated culture flasks (Sigma‒Aldrich), and cultured in plating medium (MEM) containing 10% FBS (Gibco, Carlsbad, CA, USA), 2 mM glutamine, and 1% penicillin‒streptomycin for 1 week at 37 °C in a 5% CO_2_ incubator. Microglia were removed by tapping flasks and washing twice with PBS, and astrocytes were incubated in growth media containing 5% FBS for 1 week. The medium was changed every 2–3 d.

### Iron measurements

Total iron contents in brain tissues were determined using an Iron Assay Kit (Abcam, Cambridge, MA, USA) according to the manufacturer’s instructions. Briefly, cortical penumbras, cortical cores, and striata were collected after 1, 3, 6, 12, 24, 48, 72, or 96 h of reperfusion after MCAO (60 min). Collected tissues were homogenized in a 5× volume of iron assay buffer on ice and centrifuged (13,000 × *g*, 10 min) at 4 °C. The supernatant was collected, and 5 μl of iron reducer was added to each sample and incubated for 30 min at 37 °C. Iron probe (100 μl) was added to each sample and incubated for 60 min at 37 °C away from light. Absorbance was measured at 593 nm using a microplate reader (Thermo Fisher Scientific).

### Evaluation of malondialdehyde (MDA) and 4-hydroxynonenal (4-HNE)

The MDA concentration and 4-HNE concentration in cell lysates were assessed using the Lipid Peroxidation (MDA) Assay Kit (Sigma‒Aldrich, Cat #: MAK085) and Lipid Peroxidation (4-HNE) Assay Kit (Abcam, Cat #: ab238538) according to the manufacturer’s instructions. Briefly, the MDA in the brain tissue was reacted with thiobarbituric acid (TBA) to generate the MDA-TBA adduct; the MDA-TBA adduct was quantified fluorometrically (excitation/emission = 532/553 nm) on a microplate reader.

### Perl’s Prussian blue staining

Primary neurons and microglial cells were treated with recombinant hepcidin (1, 5, or 10 ng/mL) and cultured overnight on 24-well plates on glass coverslips. Cells were then fixed with cold 4% PFA solution for 15 min, incubated with a 1:1 mixture of 1% potassium ferrocyanide and 2% hydrochloric acid for 30 min, and counterstained with 0.1% nuclear fast red for 15 min. Wells were then washed with PBS three times and mounted using mounting solution (Vector Laboratories, Peterborough, UK). Brain sections (30 μm thick) were fixed in 4% PFA solution, stained with Prussian blue solution, and counterstained with nuclear fast red solution. Prussian blue-positive cells were counted using an optical microscope (Olympus IX83, 40×; Olympus Corporation, Tokyo, Japan).

### Reverse transcription‑quantitative polymerase chain reaction (RT‑qPCR)

Total RNA was extracted from cortical cores and cortical penumbras of rat brain tissues 6, 12, or 24 h after MCAO using TRIzol reagent (Invitrogen). cDNA was synthesized using the iScript cDNA synthesis kit (Bio-RAD, Hercules, CA, USA). Target mRNA abundances were determined by real-time PCR using TOPreal qPCR 2X PreMIX SYBR Green with low ROX (Enzynomics, Daejeon, Korea). The PCR conditions used were as follows: 15 min at 95 °C, followed by 55 cycles of activation for 10 s at 95 °C and annealing/extension for 15 s at 55 °C. The primers used are listed in Table 2. PCR was performed in triplicate, and threshold cycle numbers were averaged for each sample. Ct values were normalized to those of GAPDH, and the Livak (2 − ΔΔCt) method was used to calculate changes in target gene expression.

**Table 2:** Oligonucleotide primers used for qPCR analysis.

Gene (GenBank No.)	Oligonucleotide primer sequences	PCR product size (bp)
HAMP	5′-CTATCTCCGGCAACAGACGA-3′	213
(AF344185.1)	5′-TGAGAGGTCAGGACAAGGCT-3′	
GAPDH	5′-AACCTGCCAAGTATGATGACATCA-3′	111
(BC059110.1)	5′-TGTTGAAGTCACAGGAGACAACCT-3′	
PTGS2	5′-TCACCCGAGGACTGGGCCAT-3′	135
(S67722.1)	5′-TGGGAGGATACACCTCTCCACCG-3′	
HAMP	5′-TCTCCTGCTTCTCCTCCTTG-3′	167
(NM_032541.2)	5′-AGATGCAGATGGGGAAGTTG-3′	
GAPDH	5′-AGATGCAGATGGGGAAGTTG-3′	363
(NM_001411843.1)	5′-AGATGCAGATGGGGAAGTTG-3′	

### siRNA-mediated silencing of gene expression

Primary cortical neurons and primary astrocytes were transfected with 40 pM HMGB1 siRNA and siCon (control siRNA) (both Integrated DNA Technologies, Coraville, IA, USA) or with hepcidin siRNA (Abbexa, Cambridge, UK) with 1.8 µl Oligofectamine and Opti-MEM (both Invitrogen, Carlsbad, CA, USA) for 12 h according to the manufacturer’s instructions. For in vivo experiments, 200 pM hepcidin siRNA or IL-6 siRNA (Bioneer Inc, Daejeon, Korea) was complexed with in vivo jetPEI (Polyplus-transfection, Illkirch, France) at an N/P ratio of 7, and a total volume of 20 µl was administered intranasally.

### Enzyme-linked immunosorbent assay (ELISA)

Levels of hepcidin and HMGB1 in serum and brain tissues were assayed using ELISA kits (Cusabio, Houston, TX, USA). Time-course serum sample sets were obtained by heart puncture in animals subjected to MCAO for 60 min. After whole blood collection, blood was allowed to clot by leaving it undisturbed for 20 min at room temperature. Clots were then removed by centrifuging samples at 3000 rpm for 20 min in a cold microcentrifuge. Supernatants were immediately transferred to clean polypropylene tubes, and concentrations were determined using ELISA kits. For HMGB1, tissue homogenates obtained from cortical cores and penumbras were rinsed with 1X PBS, homogenized in 1 ml (for 100 mg tissue) of 1X PBS, and centrifuged for 5 min at 5000 rpm at −4 °C. Homogenates were assayed using ELISA kits (Cusabio, Houston, TX, USA).

### Immunoblot analysis

Brain homogenates and whole cell lysates were extracted using RIPA buffer (50 mM Tris-HCl (pH 7.4), 150 mM NaCl, 1 mM EDTA, 0.5% NP40, 0.25% sodium-deoxycholate, 0.5% Triton X-100, 10% glycerol, and Complete Mini Protease Inhibitor Cocktail tablet (Roche Diagnostics, Basel, Switzerland). Cell or tissue extracts were then loaded on 10–16% SDS‒PAGE gels and immunoblotted using the following primary antibodies: anti-FPN (1:1000, Abcam), anti-DMT1 (1:3000, Abcam), anti-ferritin heavy chain (1:2000, Abcam), anti-ferritin light chain (1:2000, Abcam), anti-furin (1:3000; Bioss Antibodies), anti-hepcidin (1:2000, Bioss Antibodies), anti-SLC7A11 (1:1000, Novus Biologicals, Littleton, CO, USA), anti-GPX4 (1:1000, Abclonal Technology Woburn, MA, USA), anti-IREB2 (1:1000, HUABIO, Woburn, MA, USA), anti-α-tubulin (Santa Cruz Biotechnology), anti-GAPDH (1:5000, Cell Signaling Technology, Danvers, MA, USA) or anti-β-actin (1:3000, Santa Cruz Biotechnology). Blots were detected using anti-rabbit HP conjugated or anti-mouse HP secondary antibody (1:2000, Merck Millipore, Burlington, MA, USA) and a chemiluminescence kit (Merck Millipore).

### Immunofluorescence staining

Brains were fixed in 4% PFA solution for 2 d at 4 °C, postfixed in a 30% sucrose solution at 4 °C, sectioned at 30 μm using a vibratome, and immunologically stained. Sections were then blocked with 5% FBS, 5% horse serum, and 2% albumin in 0.1% Triton X-100 for 1 h at room temperature. Primary antibodies for goat anti-ionized calcium-binding adapter molecule-1 (Iba1) (Wako Pure Chemicals, Osaka, Japan), mouse anti-glial fibrillary acidic protein (GFAP; BD Biosciences, Franklin Lakes, NJ, USA), mouse anti-neuronal nuclei (NeuN) (Merck Millipore Corporation, Darmstadt, Germany), olig2 (R&D systems, Minneapolis, MN, USA), rabbit anti-ferritin heavy chain (Abcam), and rabbit anti-hepcidin (Bioss Antibodies) were diluted 1:200. After incubation with primary antibodies, brain sections were washed with PBS and incubated with Alexa Fluor 488-conjugated anti-goat IgG (1:300, Thermo Fisher Scientific) for anti-Iba1, rhodamine-labeled anti-mouse IgG (1:300, Merck Millipore Corporation) for anti-NeuN and anti-GFAP, Alexa Fluor 633-conjugated anti-rabbit IgG (1:200, Thermo Fisher Scientific) for anti-ferritin heavy chain, and FITC-labeled anti-rabbit IgG (1:200, Thermo Fisher Scientific) for hepcidin in PBS for 1 h at room temperature. Sections were then mounted on slides with VECTASHIELD Antifade Mounting Solution containing DAPI (Vector Laboratories) and examined under a Zeiss LSM 510 META microscope (Carl Zeiss Meditec AG, Jena, Germany).

### Treatment of primary astrocyte cultures with NMDA conditioned medium (NCM) or of primary cortical neurons with NCM-treated astrocyte culture medium (N-ACM)

Primary cortical neurons were treated with MEM (21 mM glucose) containing 300 μM NMDA (Sigma) for 30 min, washed twice with PBS, and cultured in fresh MEM for 3 h. Primary astrocyte cultures (5 × 10^5^) were then treated with this NMDA-conditioned medium (NCM) for the indicated times. Culture medium from NCM-treated astrocytes (astrocyte-conditioned medium, ACM) was collected after 6 h and added to fresh primary cortical neurons. For inhibition experiments, astrocytes were pretreated with AMD3100 (Sigma) or TLR-IN-C34 (Sigma) for 30 min before NCM treatment or NCM was preincubated with anti-HMGB1 antibody or HMGB1 A box (HM-012; HMGbiotech) for 30 min and then treated with astrocytes. For hepcidin knockdown experiments, primary astrocytes were transfected with 40 pM hepcidin siRNA (Abbexa) for 12 h before treatment with recombinant dsHMGB1 (100 ng/mL) for 6 or 12 h. Culture medium from dsHMGB1-treated astrocytes (H-ACM) was collected and added to fresh primary cortical neurons. Immunocytochemistry and immunoblot analysis were conducted as described in the previous sections.

### Statistical analysis

Two-sample comparisons were performed using Student’s *t* test, and multiple comparisons were performed using one-way or two-way analysis of variance followed by Tukey’s post hoc test. PRISM software 5.0 (GraphPad Software Inc., San Diego, CA, USA) was used for the analysis, and the results are presented as the means ± SEMs. *p* values of <0.05 were considered statistically significant.

## Results

### Rapid surge in total iron levels in postischemic brain tissues

**Fig. 1:**
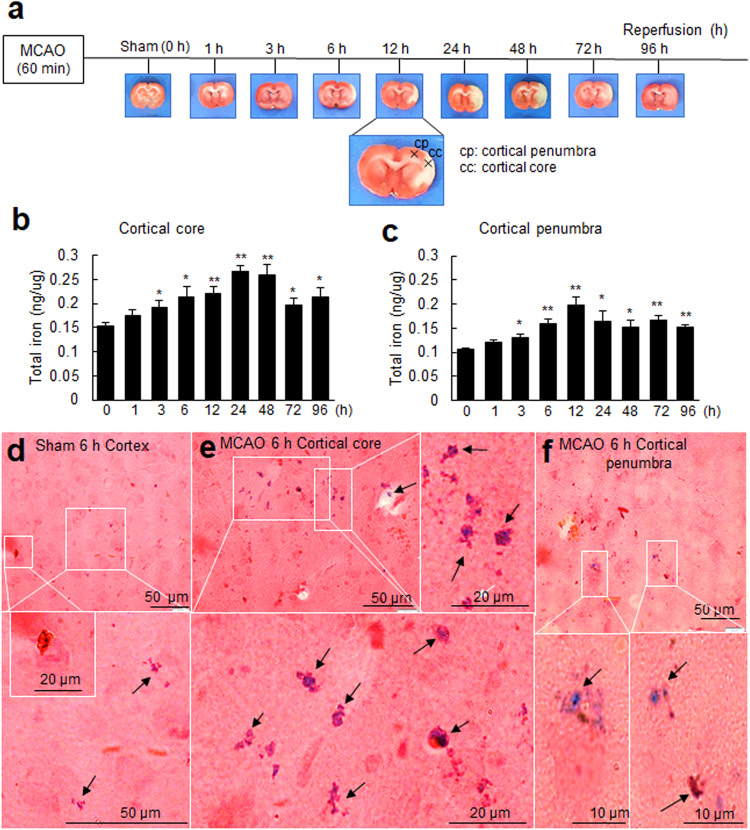
Changes in total iron levels and cellular localizations in the postischemic brain. **a** Schematic diagram presenting TTC staining showing infarct formation in the postischemic rat brain after 1, 3, 6, 12, 24, 48, 72, or 96 h of reperfusion after MCAO (60 min). Cortical core (cc) and cortical penumbra (cp) locations are presented in the lower panel. Brain tissue samples were prepared from cortical cores (**b**) and cortical penumbras (**c**) of ischemic hemispheres (as indicated in a) at 1, 3, 6, 12, 24, 48, 72, and 96 h post-MCAO. Total iron amounts were determined using an Iron Assay Kit. The results are presented as the means ± SEMs (n = 4). Coronal brain sections of the sham (**d**) and MCAO groups (**e**, **f**) at 6 h post-MCAO were stained with Perl’s Prussian blue. Representative staining in a cortical core (**e**) and cortical penumbra (**f**); Prussian blue-positive cells are indicated by arrows. The lower panels in (**d**–**f**) are high-magnification photographs of the boxed areas. The scale bars in (**d**–**f**) represent 50 µm, and those in the enlarged images represent 10, 20, or 50 µm. *p < 0.05, **p < 0.01 vs. sham-operated group.

The amounts of total iron in the brain parenchyma were examined at 1, 3, 6, 12, 24, 48, 72, and 96 h after ischemic insult in the rat MCAO model (60 min) (Fig. 1a). Total iron increased significantly in the cortical cores of ischemic hemispheres as early as 3 h post-MCAO and peaked at 24 h post-MCAO (Fig. 1b). In cortical penumbras, a significant increase was initially detected at 3 h post-MCAO, and levels peaked at 12 h. Iron levels remained elevated in cortical cores and penumbras for 96 h; however, levels at each time point were higher in cortical cores (Fig. 1b, c). Prussian blue staining showed localization of iron in the neuronal soma of cortices in sham controls (Fig. 1d). At 6 h post-MCAO, intracellular iron accumulation was detected in neurons in cortical cores and penumbras of the ischemic hemisphere (Fig. 1e, f). These results show that iron content rapidly increases in the postischemic brain.

### Local hepcidin induction in astrocytes during the acute phase after ischemic insult

**Fig. 2:**
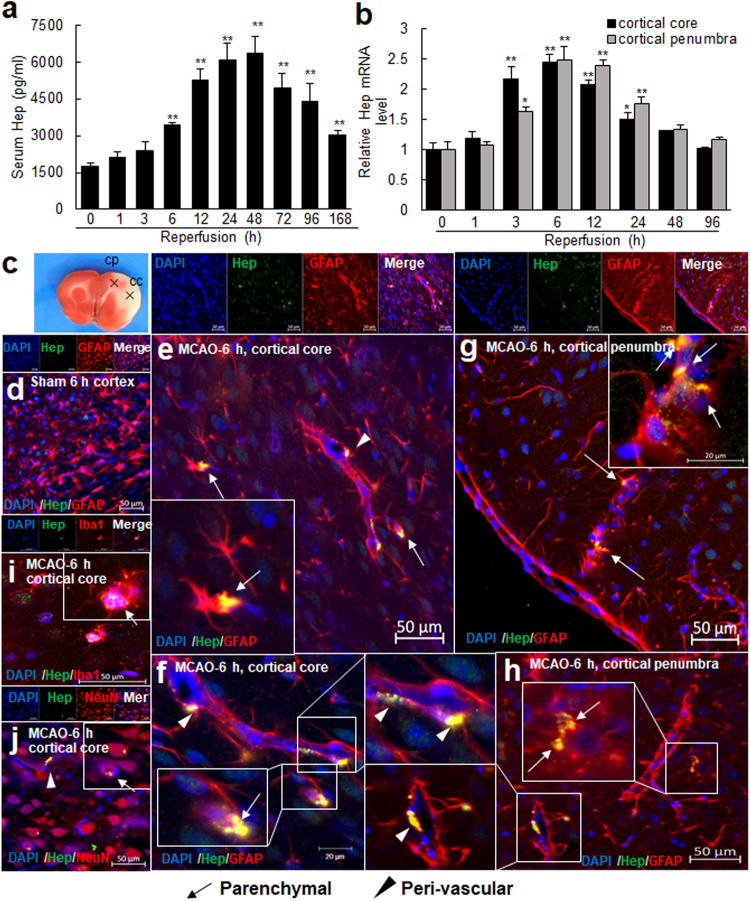
Hepcidin surge in serum and brain parenchyma after ischemic insult and hepcidin induction in astrocytes. **a** Serum hepcidin (Hep) levels were assessed by ELISA at 1, 3, 6, 12, 24, 48, 72, 96, and 168 h post-MCAO (n = 5). **b** Levels of Hep mRNA in cortical cores and cortical penumbras at 1, 3, 6, 12, 24, 48, and 96 h post-MCAO were determined by real time-quantitative PCR (RT‒qPCR). RT‒qPCR results are presented as the means ± SEMs (n = 5). *p < 0.05, **p < 0.01 vs. sham-operated groups. **c** Cortical core (cc) and cortical penumbra (cp) locations. Coronal brain sections were prepared from sham controls (**d**) and the MCAO (**e**–**j**) group at 6 h post-MCAO. Triple immunofluorescence staining was conducted with anti-Hep, anti-GFAP, and DAPI (**d**–**h**); anti-Hep, anti-Iba-1, and DAPI (**i**); or anti-Hep, anti-NeuN, and DAPI (**j**). Representative images of cortical cores (**e**, **f**, **i**, and **j**) and cortical penumbras (**g**, **h**). Arrows indicate immunoreactivity of anti-Hep in parenchyma, and arrowheads indicate immunoreactivity of anti-Hep in the peri-vascular region. The insets are high-magnification photographs. Scale bars represent 20 or 50 µm.

The observed early iron surge in the brain parenchyma prompted us to examine whether local hepcidin induction occurs in the postischemic brain during this period. Serum hepcidin levels increased rapidly from 6 h post-MCAO and peaked at 48 h, and the enhanced level was sustained until 7 d post-MCAO (Fig. 2a). In brain tissues, hepcidin upregulation occurred much earlier than that in serum; that is, it began 3 h post-MCAO in cortical cores and penumbras, peaked at 6–12 h, and then gradually decreased to the basal level at 48 h post-MCAO (Fig. 2b). Triple fluorescent immunohistochemical staining revealed hepcidin immunoreactivity in sham controls (Fig. 2d) and it was enhanced mainly in astrocytes (GFAP-positive cells) in cortical cores (Fig. 2c, e, f, i, and j) and penumbras (Fig. 2c, g, h) at 6 h post-MCAO. Hepcidin-positive astrocytes were detected in the parenchyma (Fig. 2e–g, arrows) and the perivascular region (Fig. 2e–h, arrowheads). Hepcidin induction was also detected in microglia (Iba1-positive cells) (Fig. 2i) and neurons (NeuN-positive cells) (Fig. 2j). However, the numbers of hepcidin-positive microglia and neurons were small, and the staining intensities were weaker than those observed in astrocytes. Together, these results indicate that local hepcidin expression is rapidly upregulated in the postischemic brain and that astrocytes are mainly responsible.

### Changes in the expression of iron-related molecules in postischemic brains

**Fig. 3:**
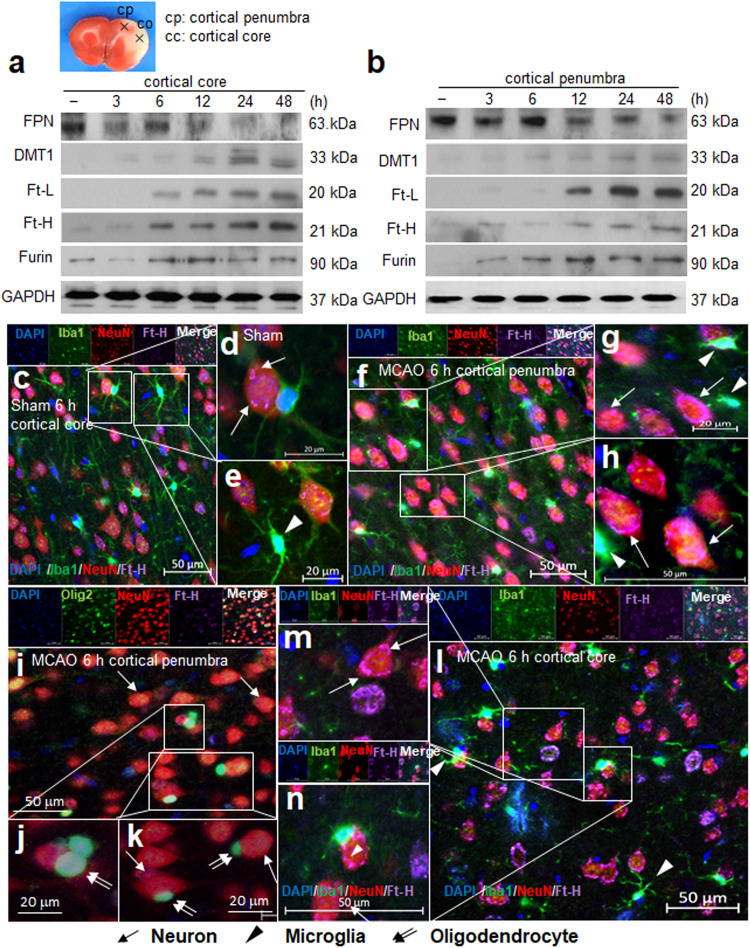
Changes in the expression of iron-related molecules in postischemic brains and the induction of ferritin heavy chain in neurons and microglia. Protein samples were prepared from cortical cores (**a**) and the penumbras (**b**) of ischemic hemispheres at 3, 6, 12, 24, and 48 h post-MCAO. Levels of ferroportin (FPN), DMT1, Ft-L, Ft-H, and Furin were assessed by immunoblotting. Coronal brain sections were prepared from sham control (**c**–**e**) and MCAO (**f**–**n**) groups at 6 h post-MCAO, and immunofluorescence staining was performed using anti-Iba1, anti-NeuN, anti-olig2, anti-Ft-H antibodies, and DAPI. The images shown are representative of cortical penumbras (**f**–**k**) and cortical cores (**l**–**n**). Arrows indicate Ft-H immunoreactivity in neurons, arrowheads indicate Ft-H immunoreactivity in microglia, and double arrows indicate Ft-H immunoreactivity in oligodendrocytes. The scale bars in (**c**, **f**, **h**, **i**, **l**), and n represent 50 μm, and those in (**d**, **e**, **g**, **j**, and **k**) represent 20 μm.

FPN levels in the brain tissue fell 3 h post-MCAO, and this reduction was maintained until 48 h post-MCAO (Fig. 3a, b). In contrast, the levels of DMT1 (the iron importer), ferritin heavy chain (Ft-H), ferritin light chain (Ft-L), and furin (prohormone convertase) increased progressively and significantly (Fig. 3a, b). Interestingly, in the core and penumbra, the initial induction and peak times differed. More specifically, FPN levels decreased and DMT1, Ft-H, Ft-L, and furin levels increased more rapidly in cortical cores than penumbras (Fig. 3a, b). Immunofluorescence staining with anti-Ft-H (a surrogate for intracellular iron) antibody showed Ft-H immunoreactivity in sham controls in neurons and microglia (Fig. 3c–e). At 6 h post-MCAO, marked Ft-H induction was observed in penumbras mainly in neurons as small dots or perinuclear rings (Fig. 3f–k). Ft-H staining was also detected in microglia (Fig. 3f–h) and in oligodendrocytes (olig 2-positive cells) (Fig. 3i–k). Ft-H induction was also clearly visible in neuron and microglial cytoplasm in cortical cores (Fig. 3l–n). These results showed that dynamic regulation of iron-modulatory proteins occurs in the postischemic brain and that neurons and microglia are the main cell types responsible.

### HMGB1 induced hepcidin upregulation in astrocytes

**Fig. 4:**
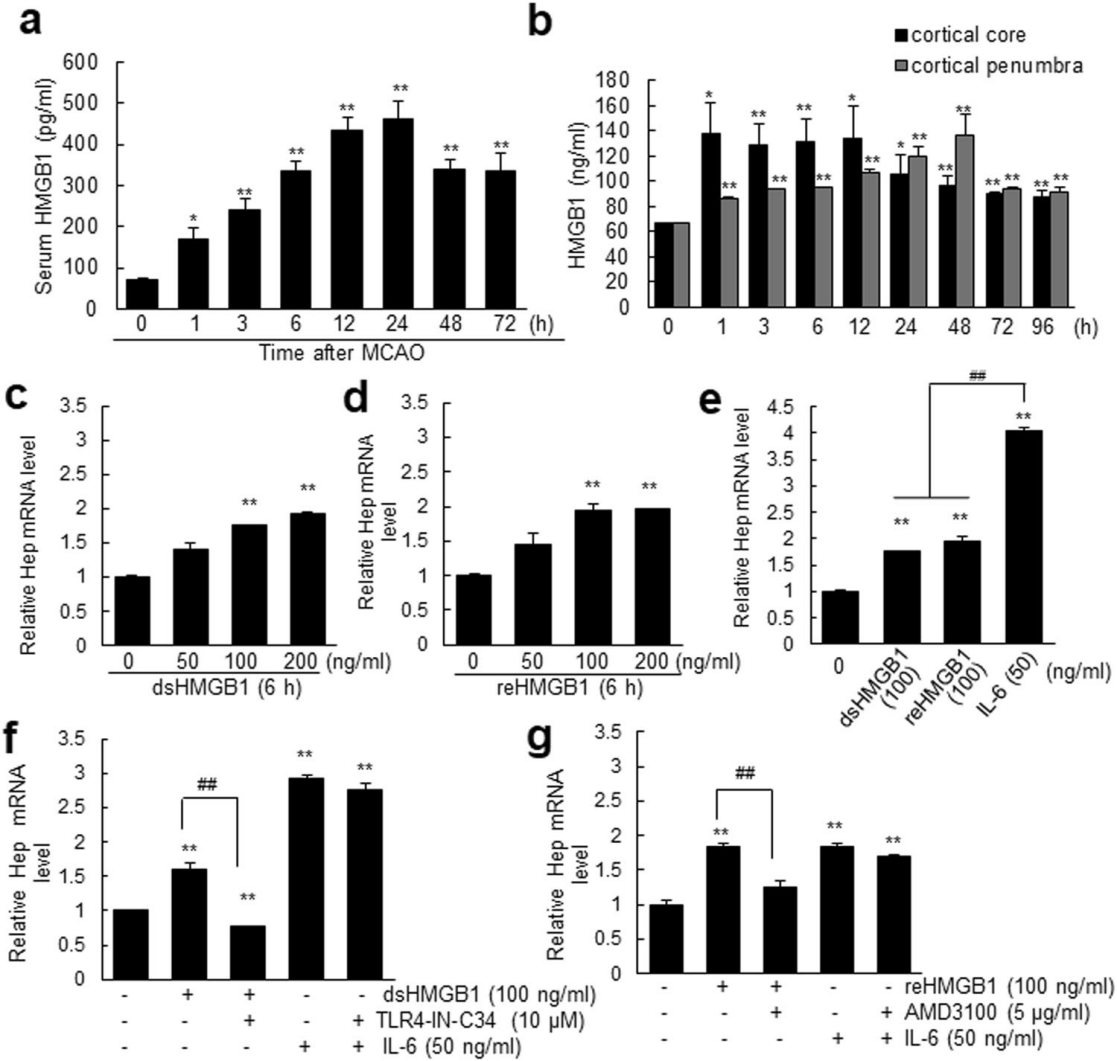
HMGB1 upregulated hepcidin expression in astrocytes. **a** Serum HMGB1 levels were assessed in rats at 1, 3, 6, 12, 24, 48, and 72 h post-MCAO by ELISA (n = 4). **b** Levels of HMGB1 in cortical cores and penumbras at 1, 3, 6, 12, 24, 48, 72, and 96 h post-MCAO were determined by ELISA (n = 4). **c**–**e** Hepcidin expression was determined by RT‒qPCR after incubating primary astrocyte cultures with dsHMGB1 (50, 100, or 200 ng/mL) (**c**), reHMGB1 (50, 100, or 200 ng/mL) (**d**), or dsHMGB1 (100 ng/mL), reHMGB1 (100 ng/mL), and IL-6 (50 ng/mL) (**e**) for 6 h. Primary astrocyte cultures were incubated with dsHMGB1 (100 ng/mL) or IL-6 (50 ng/mL) in the presence or absence of TLR4-IN-C34 (10 µM) (**f**) or with reHMGB1 (100 ng/mL) or IL-6 (50 ng/mL) in the presence or absence of AMD3100 (10 µM) (**g**) for 6 h. Hep mRNA levels were determined by RT‒qPCR. The results are presented as the means ± SEMs (n = 4), *p < 0.05, **p < 0.01 vs. sham controls, ^##^*p* < 0.01 between indicated groups.

When we examined serum HMGB1 levels during the acute to subacute period after ischemic insult using ELISA, significant HMGB1 accumulation in serum was observed as early as 1 h post-MCAO and then rapidly increased until 24 h post-MCAO (Fig. 4a). An acute surge of HMGB1 also occurred in the brain parenchyma in both the cortical cores and penumbras from 1 h and was much higher in the core than in the penumbra, with significantly elevated levels of HMGB1 maintained for 96 h (Fig. 4b). To determine whether this accumulated HMGB1 could upregulate hepcidin in astrocytes, we examined hepcidin expression in primary astrocytes after treating them with recombinant HMGB1. RT‒qPCR showed that hepcidin was significantly upregulated in dsHMGB1 (100 ng/mL)- and reHMGB1 (100 ng/mL)-treated astrocytes (Fig. 4c, d) and that these upregulations were slightly lower than that caused by IL-6 (50 ng/ml), a well-known inducer of hepcidin in astrocytes^33,35^ (Supplementary Fig. 1) (Fig. 4e). Furthermore, dsHMGB1- and reHMGB1-mediated hepcidin inductions were significantly suppressed by cotreating astrocytes with TLR4-IN-C34 (an inhibitor of TLR4, 10 μM) or with AMD3100 (antagonist of CXCR4, a ligand for reHMGB1, 5 μg/mL), respectively (Fig. 4f, g). However, neither TLR4-IN-C34 nor AMD3100 inhibited IL-6-mediated hepcidin upregulation (Fig. 4f, g). Collectively, these results show that dsHMGB1 and reHMGB1 can upregulate hepcidin in astrocytes and that endogenous TLR4 and CXCR4, respectively, are involved in these upregulations.

### Hepcidin induced ferroptosis in neurons by decreasing FPN and increasing iron-related proteins and intracellular iron

**Fig. 5:**
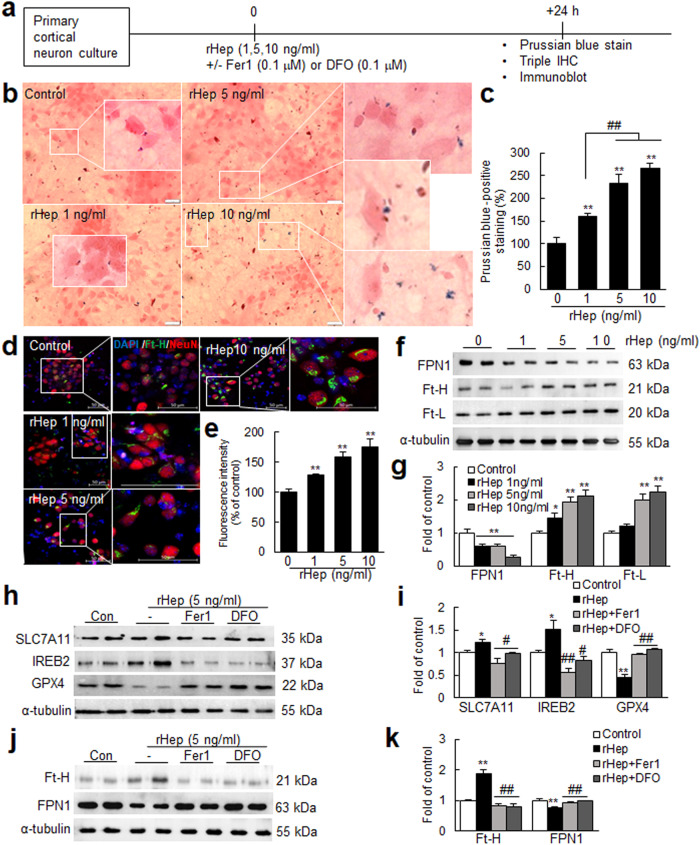
Hepcidin upregulated intracellular iron levels in neurons and modulated FPN and iron-related protein levels. (**a**) Primary cortical neuron cultures were treated with recombinant hepcidin (rHep; 1, 5, or 10 ng/mL) for 24 h. **b**, **c** Neurons were stained with Prussian blue and nuclear fast red (a nuclear counterstain). Representative images are presented in (**b**), and Prussian blue-positive staining results are presented as the means ± SEMs (n = 12) in (**c**). **d**, **e** Triple immunofluorescence staining was carried out with anti-Ft-H antibody, anti-NeuN antibody, and DAPI. The right-side panels in (**d**) are higher magnifications of the boxed regions. **f**, **g** Levels of FPN, Ft-L, and Ft-H were assessed by immunoblotting. rHep (5 ng/mL) was cotreated with Fer-1 (0.1 μM) or DFO (0.1 μM) for 24 h, and the levels of SLC7A11, IREB2, GPX4 (**h**, **i**) and FPN1 and Ft-H (**j**, **k**) were assessed by immunoblotting. Representative images are shown in (**d**, **f**, **h**, and **j**), and the results are presented as the means ± SEMs (n = 4) in (**e**, **g**, **i** and **k**). Scale bars represent 50 μm. *p < 0.05, **p < 0.01 vs. controls and ^#^*p* < 0.05, ^##^*p* < 0.01 between indicated groups.

To investigate whether hepcidin induces iron accumulation in neurons, primary cortical neuron cultures were treated with recombinant hepcidin (rHep, 1, 5, or 10 ng/mL) and then stained with Perl’s Prussian blue (Fig. 5a). Iron was detected in PBS-treated control cells as blue dots (Fig. 5b), and rHep treatment significantly increased the numbers and sizes of the dots in a dose-dependent manner (Fig. 5b, c). Triple fluorescence staining with anti-NeuN antibody, anti-Ft-H antibody, and DAPI showed that rHep significantly induced Ft-H immunoreactivity in neurons (Fig. 5d, e). In addition, rHep dose-dependently downregulated FPN and upregulated Ft-H and Ft-L (Fig. 5f, g). Recombinant hepcidin (1, 5, or 10 ng/mL)-induced iron accumulation and down- and upregulation of FPN and DMT1, Ft-H and Ft-L, respectively, were also observed in primary microglia cultures (Supplementary Fig. 2). Next, we examined whether this iron accumulation results in ferroptosis in neurons. The expression levels of solute carrier family 7 member 11 (SLC7A11) and iron responsive element binding protein 2 (IREB2), known to be induced during ferroptosis following CNS injury^45–47^, were significantly increased in rHep-treated cortical neuron cultures; in contrast, the level of glutathione peroxidase 4 (GPX4), another ferroptosis marker^46,47^, was significantly decreased (Fig. 5h, i). Importantly, cotreatment with ferrostatin-1 (Fer-1, a ferroptosis inhibitor, 0.1 μM)^47^ significantly mitigated all up- and downregulation of ferroptosis markers (Fig. 5h–k), and similar inhibition, including FPN1 downregulation and Ft-H upregulation, was also observed after cotreatment with deferoxamine (DFO; an iron chelator, 0.1 μM) (Fig. 5h–k). Taken together, these results demonstrate that hepcidin induces iron accumulation and subsequent ferroptosis in neurons.

### HMGB1 released from neurons induced hepcidin in astrocytes, leading to iron accumulation and ferroptosis in neurons

**Fig. 6:**
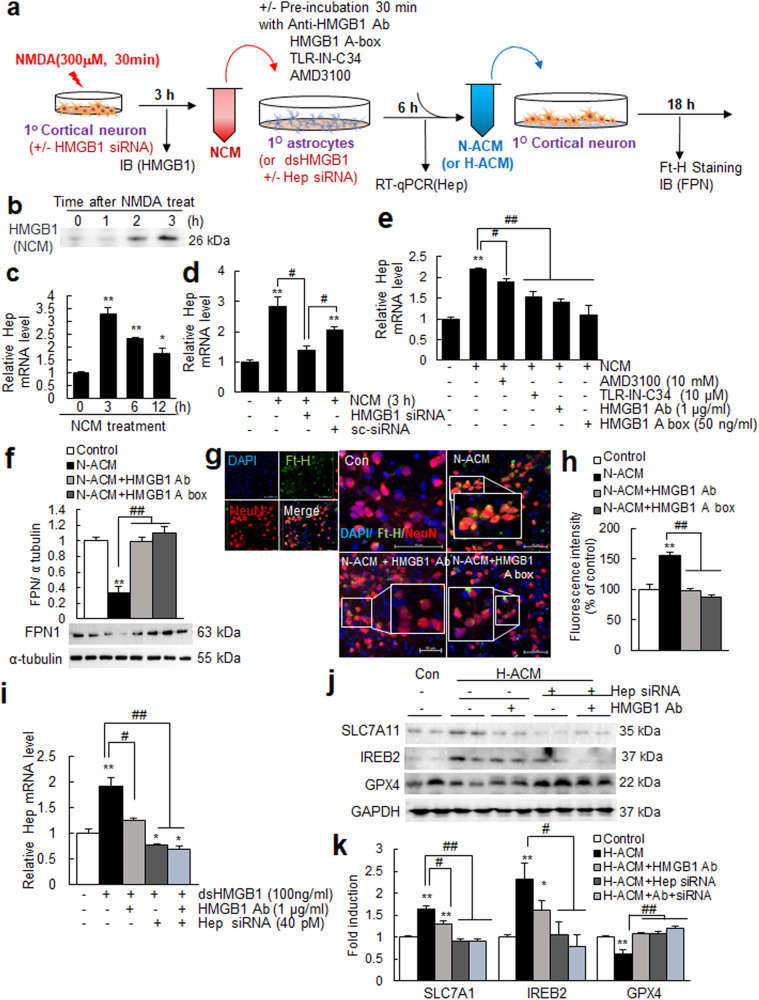
NMDA-conditioned media increased hepcidin levels in astrocytes and iron accumulation in neurons. **a** Schematic diagram of the procedure used NMDA-conditioned media (NCM) and NCM-treated astrocyte conditioned media (N-ACM). **b** HMGB1 levels in the NCM of primary cortical neurons (1.6 × 10^6^/4-well) were examined by immunoblotting after 1, 2, or 3 h of NMDA treatment (300 μM, 30 min). **c** Primary astrocyte cultures (5 × 10^5^/well) were treated with NCM for 1, 2, or 3 h, and hepcidin levels were determined by RT‒qPCR. **d** NCM was prepared after transfecting HMGB1 siRNA or scrambled siRNA (sc-siRNA) and treating primary astrocyte cultures for 3 h, and hepcidin levels were determined by RT‒qPCR. **e** Primary astrocyte cultures (5 × 10^5^/well) were preincubated with AMD3100 (10 μM) or TLR4-IN-C34 (10 μM) for 30 min and then treated with NCM or incubated with NCM, which was obtained from primary cortical neurons preincubated with anti-HMGB1 antibody (1 μg/ml) or HMGB1 A box (50 ng/ml) for 30 min and then treated with NMDA. Hepcidin expression levels in primary astrocyte cultures (5 × 10^5^/well) were determined by RT‒qPCR after 6 h of NCM incubation. **f**–**h** NCM-conditioned astrocyte culture media (N-ACM) were prepared as described in (**c**), and naïve primary cortical cultures (4 × 10^5^/well) were incubated with this N-ACM for 18 h. The levels of FPN and Ft-H were assessed by immunoblotting (**f**) and immunofluorescence staining (**g** and **h**), respectively. **i**–**k** Primary astrocytes were transfected with hepcidin siRNA (40 pM) 3 h before treatment with recombinant dsHMGB1 (100 ng/ml). Conditioned media were prepared 6 h after treatment with recombinant dsHMGB1 (H-ACM), treated with naïve primary cortical neuron cultures (4 × 10^5^/well) and incubated for 18 h. Hepcidin expression levels in primary astrocyte cultures were determined by RT‒qPCR after 6 h of NCM incubation (**i**), and the levels of SLC7A11, IREB2, and GPX4 in H-ACM-treated primary cortical neurons were assessed by immunoblotting. Representative images are presented in (**f**, **g**, and **j**), and the results are presented as the means ± SEMs (n = 4 or 12). Scale bars represent 50 μm. *p < 0.05, **p < 0.01 vs. controls and ^#^*p* < 0.05, ^##^*p* < 0.01 between indicated groups.

To confirm the role played by HMGB1 and investigate the interplay between brain cells, we conducted a series of experiments using conditioned media (Fig. 6a). When primary cortical neuron cultures were treated with N-methyl-D-aspartate (NMDA; 300 μM, 30 min), HMGB1 accumulated in NMDA-conditioned media (NCM) (Fig. 6b). Furthermore, treatment of primary astrocyte cultures with NCM collected after 3 h of NMDA treatment significantly increased hepcidin synthesis in astrocytes (Fig. 6c). However, astrocytes failed to induce hepcidin when treated with NCM prepared from neurons after HMGB1 knockdown using HMGB1 siRNA (Fig. 6d), indicating that neuronal HMGB1 plays a key role. This NCM-mediated hepcidin upregulation was significantly suppressed by preincubating NCM with anti-HMGB1 antibody or HMGB1 A box (a well-known functional inhibitor of HMGB1) or by preincubating astrocytes with TLR4-IN-C34 or AMD3100 before NCM treatment (Fig. 6e), which further confirmed an important role of HMGB1 in NCM-mediated hepcidin upregulation in astrocytes. Moreover, when naïve primary cortical neurons were treated with NCM-treated astrocyte-conditioned media (N-ACM) for 18 h, FPN was downregulated, and the amount of Ft-H was increased; however, these up- and downregulations were suppressed when naïve primary cortical neurons were incubated with N-ACM together with anti-HMGB1 antibody or HMGB1 A box (Fig. 6f–h). All these results confirmed that HMGB1 played a critical role in N-ACM-induced iron upregulation in neurons. Next, we investigated the role of hepcidin induction in astrocytes. As shown in Fig. 4c, significant hepcidin induction was detected in astrocytes after treatment with dsHMGB1 (100 ng/mL) but not after hepcidin knockdown in astrocytes (Fig. 6i). When naïve primary cortical neurons were treated with astrocyte-conditioned media prepared from dsHMGB1-treated astrocytes (H-ACM), significant up- or downregulation of ferroptosis markers was detected (Fig. 6j, k). However, it was not detected in H-ACM-treated neurons when H-ACM was prepared from astrocytes after hepcidin knockdown (Fig. 6j, k). The results indicate that astrocyte hepcidin plays a central role in inducing ferroptosis in neurons. It is important to note here that in the absence of astrocyte hepcidin, no inhibitory effects of anti-HMGB1 Ab on ferroptosis induction were observed (Fig. 6j, k), demonstrating a hepcidin-dependent effect of HMGB1 Ab. Together, these results indicate that HMGB1 plays a critical role in hepcidin induction in astrocytes and subsequent ferroptosis induction in neurons.

### Ferroptosis occurred early in the postischemic brain

**Fig. 7:**
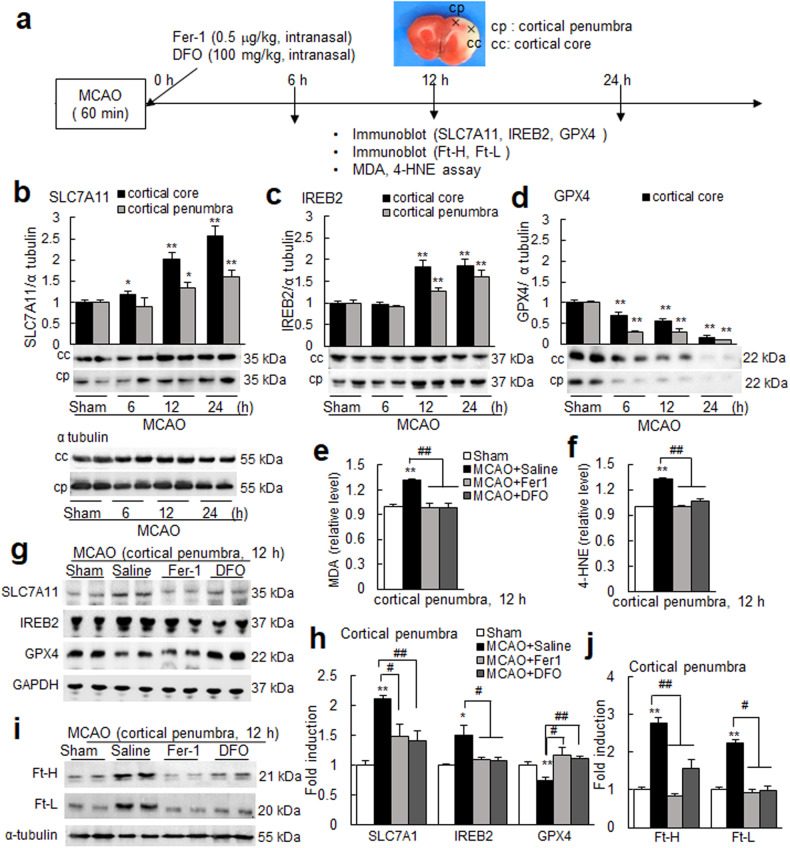
Ferroptosis in the postischemic brain. **a** Levels of SLC7A11, IREB2, GPX4, MDA, and 4-HNE were examined after intranasal administration or not of ferrostatin-1 (Fer-1, 0.5 mg/kg) or deferoxamine (DFO, 100 mg/kg) immediately after suture removal. Locations of the cortical core (cc) and cortical penumbra (cp) are presented. **b**–**d** Levels of SLC7A11, IREB2, and GPX4 in cortical cores or in the cortical penumbras of ischemic hemispheres at 6, 12, or 24 h post-MCAO were determined by immunoblotting. **e**, **f** Levels of MDA and 4-HNE at 12 h post-MCAO were determined by assay kits. **e**–**j** Fer-1 (0.5 mg/kg) or DFO (100 mg/kg) was administered intranasally immediately after suture removal. Levels of MDA, 4-HNE, SLC7A11, IREB2, GPX4, Ft-H, and Ft-L in cortical penumbras of ischemic hemispheres at 12 h post-MCAO were determined. Representative images are presented in (**b**–**d**, **g**, and **i**), and quantified results are presented as the means ± SEMs (n = 4). Sham, sham-operated animals (n = 4); MCAO+saline, saline-administered MCAO animals (n = 4); MCAO+Fer-1, Fer-1-administered MCAO animals (n = 4); MCAO + DFO, DFO-administered MCAO animals (n = 4). *p < 0.05, **p < 0.01 vs. sham-operated group and ^#^*p* < 0.05, ^##^*p* < 0.01 between indicated groups.

Neuronal ferroptosis observed by N-ACM prompted us to investigate whether ferroptosis occurs in the ischemic brain (Fig. 7a). The expression levels of SLC7A11 and IREB2 were significantly increased in the cortical penumbras of ischemic hemispheres, and the levels of GPX4 were significantly decreased (Fig. 7b–d). Furthermore, the amounts of malondialdehyde (MDA) and 4-hydroxynonenal (4-HNE) were also significantly increased at 12 h post-MCAO (Fig. 7e, f). Importantly, the administration of Fer-1 (0.5 mg/kg)^47^ or DFO (100 mg/kg) immediately after MCAO (Fig. 7a) significantly suppressed MDA and 4-HNE induction (Fig. 7e, f) and up- or downregulated ferroptosis markers (Fig. 7g, h). Suppression of enhanced iron levels (measured by iron level surrogates, Ft-H and Ft-L) was also observed after Fer-1 or DFO administration (Fig. 7i, j). Furthermore, similar suppression was also detected in cortical cores (Supplementary Fig. 3). Taken together, these results show that ferroptosis occurs in the postischemic brain.

### Blocking HMGB1 function with an anti-HMGB1 antibody or HMGB1 A box suppressed ferroptosis in the postischemic brain

**Fig. 8:**
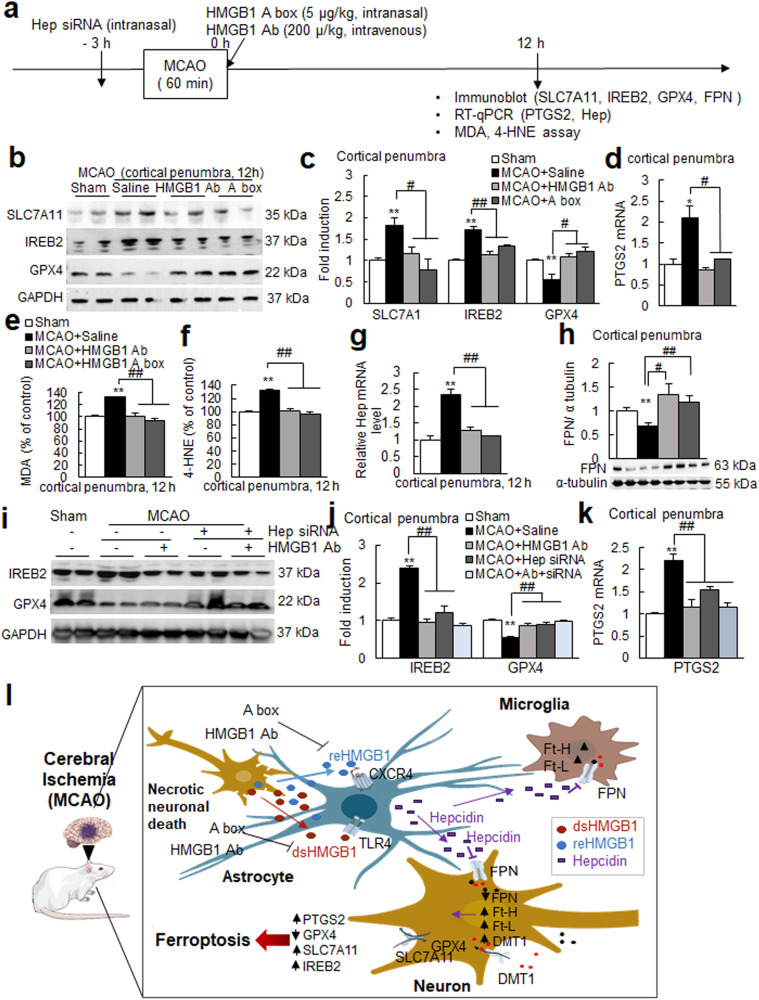
Blocking HMGB1 function with an anti-HMGB1 antibody or HMGB1 A box suppressed ferroptosis in the postischemic brain. **a** HMGB1 A box (5 µg/kg) or anti-HMGB1 antibody (200 µg/kg) was administered intranasally and intravenously, respectively, immediately after suture removal. **b**–**d** Levels of SLC7A11, IREB2, GPX4, and PTGS2 in cortical penumbras of ischemic hemispheres at 12 h post-MCAO were determined by immunoblotting (**b**, **c**) or RT‒qPCR ((**d**) for PTGS2). Representative images are presented in b, and the results are presented as the means ± SEMs (n = 4) in (**c**). **e**, **f** Levels of MDA and 4-HNE in the cortical penumbras of ischemic hemispheres at 12 h post-MCAO were determined by assay kits. **g**, **h** Levels of Hep and FPN in the cortical penumbras of ischemic hemispheres at 12 h post-MCAO were determined by RT‒qPCR and immunoblotting, respectively. **i**–**k** Hepcidin siRNA (200 pM) was administered intranasally 3 h before MCAO, and anti-HMGB1 antibody (200 µg/kg) was administered intravenously immediately after suture removal. Levels of IREB2, GPX4, and PTGS2 in the cortical penumbras of ischemic hemispheres at 12 h post-MCAO were determined by immunoblotting (**i**, **j**) or RT‒qPCR ((**k**) for PTGS2). Sham, sham-operated animals (n = 4); MCAO+Saline, saline-administered MCAO animals (n = 4); MCAO + HMGB1 Ab, anti-HMGB1 Ab-administered MCAO animals (n = 4); MCAO + HMGB1 A box, HMGB1 A box-administered MCAO animals (n = 4); MCAO+Hep, hepcidin siRNA-administered MCAO animals (n = 4). *p < 0.05, **p < 0.01 vs. sham-operated group and ^#^*p* < 0.05, ^##^*p* < 0.01 between indicated groups. **l** Diagram showing interplay between brain cells during hepcidin induction and iron surge in the postischemic brain and the role of HMGB1. HMGB1 is released from neurons and glia during the acute phase of cerebral ischemia and upregulates hepcidin expression in astrocytes. Enhanced local hepcidin levels downregulated ferroportin and upregulated the levels of iron-related proteins in neurons and microglia, which, in turn, induced intracellular iron accumulation and ferroptosis in the postischemic brain. dsHMGB1 disulfide high mobility group Box 1; reHMGB1 reduced high mobility group Box 1; Hep hepcidin; FPN ferroportin; TLR4 Toll-like receptor; CXCR4 C-X-C chemokine receptor type 4; Ft-H ferritin heavy chain; Ft-L ferritin light chain; DMT1 divalent metal transporter 1; SLC7A11 cystine-glutamate antiporter; GPX4 glutathione peroxidase 4; IREB2 iron responsive element binding protein 2; PTGS2 prostaglandin-endoperoxide synthase 2.

When we inhibited HMGB1 function by intranasal administration of the HMGB1 A box immediately after MCAO (Fig. 8a), the inductions of SLC7A11, IREB2, and prostaglandin-endoperoxide synthase 2 (PTGS2) and the reduction of GPX4 observed in cortical penumbras at 12 h post-MCAO were all suppressed, and similar suppressions were observed after anti-HMGB1 antibody administration (Fig. 8b–d). In addition, the inductions of MDA and 4-HNE observed at 12 h post-MCAO were also significantly suppressed by anti-HMGB1 antibody or HMGB1 A box (Fig. 8e, f). Importantly, hepcidin upregulation and FPN downregulation observed in cortical penumbras at 12 h post-MCAO were significantly suppressed in the MCAO + HMGB1 A box and MCAO+anti-HMGB1 antibody groups (Fig. 8g, h). Similar reversions were also observed in the cortical core (Supplementary Fig. 4). It is interesting to note here that hepcidin upregulation was suppressed significantly after knocking down IL-6, and this suppressed level was further reduced significantly to near-basal levels by coadministration of anti-HMGB1 Ab (Supplementary Fig. 5), indicating a critical contribution of HMGB1 to hepcidin upregulation in the postischemic brain in the absence of IL-6. Moreover, in the hepcidin knockdown condition (via intranasal administration of hepcidin siRNA 3 h before MCAO), the up- or downregulation of ferroptosis markers was significantly suppressed (Fig. 8i–k), indicating a critical role of hepcidin in ferroptosis induction in the postischemic brain. Importantly, no further inhibition by the anti-HMGB1 Ab was detected in the absence of hepcidin (Fig. 8i–k), confirming a hepcidin-dependent function of HMGB1. However, mild but significant suppression of HMGB1 induction at 12 h post-MCAO (Supplementary Fig. 6) and smaller infarct volumes (Supplementary Fig. 7) in the MCAO + HMGB1 A box and MCAO+anti-HMGB1 antibody groups compared to the MCAO control group suggest that we cannot exclude the possibility of an indirect protective effect of anti-HMGB1 antibody or HMGB1 A box. Together, these results indicate that functional blocking of HMGB1 suppresses ferroptosis probably by suppressing HMGB1-mediated hepcidin induction and the subsequent downregulation of FPN in the postischemic brain.

## Discussion

Dysregulation of iron levels in the brain is strongly associated with poor outcomes under numerous pathological conditions, and thus, a means of regulating brain iron levels is needed. Reports of no significant alterations in brain iron depots despite systemic iron deficiency^48^ or in systemic (liver) hepcidin knockout mice^25^ support this notion. The present study shows that HMGB1 released by damaged neurons during the acute phase of cerebral ischemia induces the upregulation of hepcidin in astrocytes, which in turn induces intracellular iron accumulation in neurons and causes ferroptosis in the postischemic brain (Fig. 8l). Although the regulation of intracellular iron levels via local hepcidin expression has been reported in various pathological conditions^49,50^, this report is the first to describe the molecular mechanism underlying the local hepcidin induction-mediated iron surge and subsequent ferroptosis in the ischemic brain and to propose that HMGB1 acts as a key modulator.

The current study describes the temporal kinetics of local hepcidin upregulation, iron surge, and ferroptosis induction in the postischemic brain, especially during the acute to subacute phase. In terms of interplay among various brain cells during this process, components that participate in the local iron surge in the ischemic brain were detected sequentially in neurons, astrocytes, and microglia. In addition, in general, local iron surge and associated changes occurred earlier and at higher levels in the ischemic core than in the penumbra. We suppose that this may have been due to more severe damage in the core region, resulting in rapid HMGB1 release (Fig. 4b) and the triggering of local hepcidin production during the acute period. It is important to note that the kinetics of serum hepcidin induction differed compared to the brain, that is, a delayed start at 6 h, a higher peak at 48 h, and sustained induction until 7 days post-MCAO, which indicated that systemic hepcidin upregulation, for example, by hepatocytes, occurs at later times.

dsHMGB1 and reHMGB1 were found to have comparable hepcidin-inducing capacities in astrocytes. However, they were reported to play differential roles under pathological conditions; for example, dsHMGB1 stimulates microglia to exacerbate the proinflammatory response via the TLR4 signaling pathway^39–41^. Moreover, TLR4 mediates hepcidin upregulation in LPS-treated hepatocytes^42^ and retinal pigment epithelium^18^, and TLR4 activation under inflammatory conditions can directly suppress cellular FPN mRNA levels and decrease cellular iron export to blood plasma^51^. Therefore, we cannot exclude the possibility that dsHMGB1-TLR4 makes a greater contribution than reHMGB1 to hepcidin induction in the postischemic brain, and further studies are required to determine the relative amounts of these two HMGB1 types in the brain during the critical period. In addition, although the hepcidin-inducing capacities of the two HMGB1 types were found to be less than that of IL-6, the detection of significant hepcidin induction in the cortex and hippocampus of LPS-treated IL-6 knockout mice^52^ indicates that IL-6-independent hepcidin upregulation occurs in the brain under inflammatory conditions. In the current study, we also showed hepcidin upregulation in the postischemic brain after knocking down IL-6, and it was suppressed significantly by coadministration of anti-HMGB1 Ab (Supplementary Fig. 5), indicating that HMGB1 contributes to hepcidin upregulation in the postischemic brain in the absence of IL-6. Thus, both IL-6 and HMGB1 are involved in hepcidin upregulation. The importance of HMGB1 and other factors, including IL-6, with respect to hepcidin induction after cerebral ischemia requires further study.

In the postischemic brain, FPN levels gradually but significantly decreased, which suggests that the downregulation of this iron exporter might be responsible for the acute iron surge observed in the brain parenchyma. The higher density of FPN in neurons than in other brain cells could explain, in part, why hepcidin-mediated FPN downregulation results in a higher intracellular iron load in neurons^33,53^. However, hepcidin-mediated FPN downregulation and Ft-H upregulation also occurred in microglia (Supplementary Fig. 2). Interestingly, hepcidin-mediated FPN internalization was reported to underlie iron accumulation in microglia in an autoimmune encephalomyelitis mouse model^54^. In addition, iron stored in microglia is released by noggin and promotes myelin repair in the ischemic brain^55^. Moreover, under iron overload conditions in the brain, microglia increase ferritin expression, thereby contributing to the sequestration and storage of excess iron^56^. Similarly, oligodendrocytes^56^ and astrocytes^57^ are reported to sequester and store iron. These observations suggest that hepcidin-mediated changes in intracellular iron levels in glial cells might protect neurons from damage. Additionally, studies are required to determine the modulatory effects of microglia and oligodendrocytes on neuronal iron status in the postischemic brain.

In the present study, we found that ferroptosis in the postischemic brain was suppressed by an anti-HMGB1 antibody or HMGB1 A box as effectively as by Fer-1 or DFO. A significant suppression of local hepcidin induction and ferroportin reduction in the postischemic brain by anti-HMGB1 antibody or HMGB1 A box administration supports this notion. Therefore, we added an anti-ferroptotic effect to the anti-inflammatory and anti-excitotoxicity neuroprotective effects^36,37,41^ conferred by inhibiting HMGB1 in the postischemic brain. Recently, the involvement of HMGB1 in ferroptosis induction was reported in leukemia^58^ and doxorubicin-induced cardiomyopathy^59^ based on the observation that HMGB1 inhibition suppressed ferroptosis. Since HMGB1 plays a critical role in excitotoxicity-induced necrosis and inflammation during the acute to subacute phases in the postischemic brain, it appears that crosstalk between ferroptosis and the processes mentioned above needs further study.

Time-dependent paracrine interactions between brain cells, such as neurons, astrocytes, microglia, and oligodendrocytes, resulting from HMGB1 secretion, hepcidin induction, FPN downregulation, and iron accumulation, were observed during iron modulation in the postischemic brain (Fig. 8l). Therefore, observations based on isolated neurons or microglial cultures cannot represent in vivo interrelationships. Furthermore, these interactions explain why isolated neurons do not exhibit hepcidin induction or FPN downregulation in response to inflammatory stimuli^35^ unless they are cocultured with microglia^60^ or treated with conditioned media obtained from IL-6-treated astrocytes^35^. Moreover, each cell type performs more than one function during iron regulation to generate a complex combination of context-specific autocrine and paracrine functions. Therefore, interpretations between participating cells must take those points into account.

In conclusion, iron metabolism, inflammation, and oxidative stress form a vicious cycle under various pathological conditions, which suggests that attenuation of the inflammatory response or pro-oxidative stress might break this vicious cycle and reduce ferroptotic brain injury in the ischemic brain. HMGB1 should be regarded as a valuable target for mitigating ischemic brain damage due to its central role in this vicious cycle.

### Supplementary information

Supplementary materials
