# Supplementary material for: HMGB1 induces hepcidin upregulation in astrocytes and causes an acute iron surge and subsequent ferroptosis in the postischemic brain
Source: Exp Mol Med. 2023 Nov 1;55(11):2402–16. doi: 10.1038/s12276-023-01111-z (PMC10689467; doi:10.1038/s12276-023-01111-z)
Supplement: Supplementary file 1 — Supplementary materials [file 12276_2023_1111_MOESM1_ESM.pdf]

Supplementary materials

HMGB1 induces hepcidin upregulation in astrocytes and causes an acute iron surge and subsequent ferroptosis in the postischemic brain

Dashdulam Davaanyam[a#], Hahnbi-Lee[a#], Song-I Seol[a], Sang-A Oh[a], Seung-Woo Kim[b], Ja-Kyeong Lee[a,*]

[a]Department of Anatomy, Inha University School of Medicine, Incheon, 22212, Korea

[b]Department of Biomedical Sciences, Inha University School of Medicine, Inchon 22212, Korea

[#] These authors are equally contributed

Key words: HMGB1; hepcidin; iron; MCAO; astrocytes

[*]Corresponding author:

Ja-Kyeong Lee, Ph.D.

Department of Anatomy, Inha University School of Medicine

Inharo 100, Inchon, 22202, Republic of Korea

Tel, +82-32-860-9893; jklee@inha.ac.kr

Materials and Methods

Staining with 2, 3, 5-triphenyl tetrazolium chloride (TTC)

Rats were sacrificed 12 h post-MCAO, and whole brains were sliced coronally at 2 mm using a metallic brain matrix (RBM-40000, ASI, Springville, UT, USA). Tissue slices were immediately incubated in saline containing TTC (2, 3, 5-triphenyl tetrazolium chloride, 2%) for 15 min at 37 °C and then stored in 4% paraformaldehyde (PFA).

Primary microglial cultures

Primary microglial cultures were prepared as described previously[36]. Briefly, cells dissociated from the cerebral hemispheres of 1-day-old postnatal rat brains (Sprague–Dawley strain) were seeded at a density of 1.2×10^6 cells/mL in Dulbecco's modified Eagle′s medium (DMEM; Gibco) containing 10% FBS (Hyclone, Logan, UT, USA) and 1% penicillin-streptomycin (Gibco BRL, Gaithersburg, MD, USA). After culture for 2 weeks, microglia were detached from flasks by mild shaking, and astrocytes were removed by filtering using a cell strainer (BD Falcon, Bedford, MA, USA). After centrifugation (1000× g) for 5 min, cells were resuspended in fresh DMEM containing 10% FBS and 1% penicillin-streptomycin, plated at a final density of 1.5×10^5 cells/well on a 24-well culture plate (Corning, Corning, NY, USA), and cultured for 2 h and the medium was changed to DMEM containing 5% FBS and 500 μM B27 (Gibco BRL).

Supplementary Figure 1

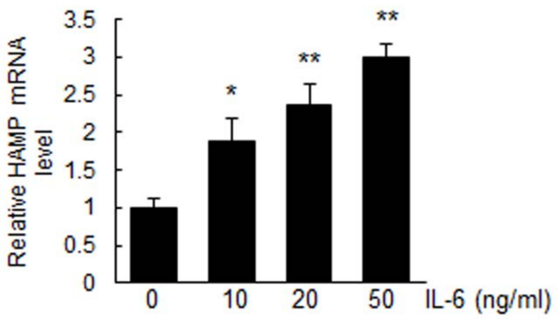

Supplementary Fig. 1. IL-6 induces hepcidin expression in astrocytes in a dose-dependent manner

Primary astrocyte cultures were incubated with IL-6 (10, 20, or 50 ng/mL) for 6 h, and hepcidin levels were determined by RT-qPCR. The results are presented as means ± SEMs (n=3). *p < 0.05, **p < 0.01 vs. control groups.

Supplementary Figure 2

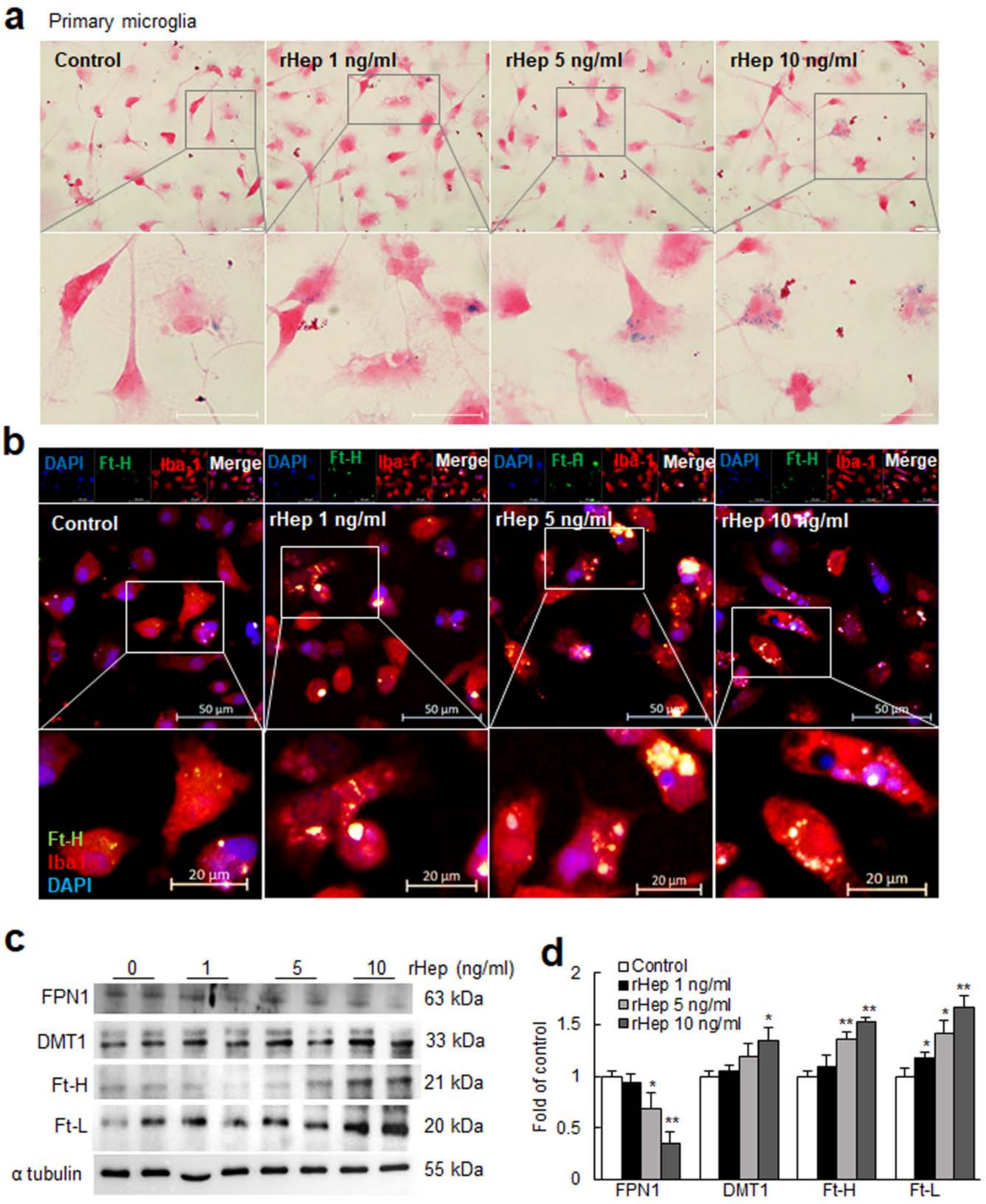

Supplementary Fig. 2. Hepcidin upregulates intracellular iron levels in microglia and modulates levels of FPN and iron-related proteins

Primary microglial cultures were treated with recombinant hepcidin (rHep; 1, 5, or 10 ng/mL)

for 24 h. (a) Prussian blue staining was performed, and nuclear fast red staining was carried out as a counter-staining to show the position of the nucleus. The lower panels in a show higher magnifications of the boxed regions in the upper panel. (b) Triple immunofluorescence staining was performed with an anti-ferritin heavy chain (Ft-H) antibody, anti-Iba-1 antibody, and DAPI. The lower panels in b show higher magnifications of the boxed regions in the upper panel. (c-d) Levels of FPN, DMT1, Ft-L, and Ft-H were examined by immunoblotting (α-tubulin was used as a loading control). The scale bars in the upper panels in a and b represent 50 μm, and those in the lower panels represent 20 μm. Representative images are presented in c and quantified data are presented as means ± SEMs (n=4). *p < 0.05, **p < 0.01 vs. control groups.

Supplementary Figure 3

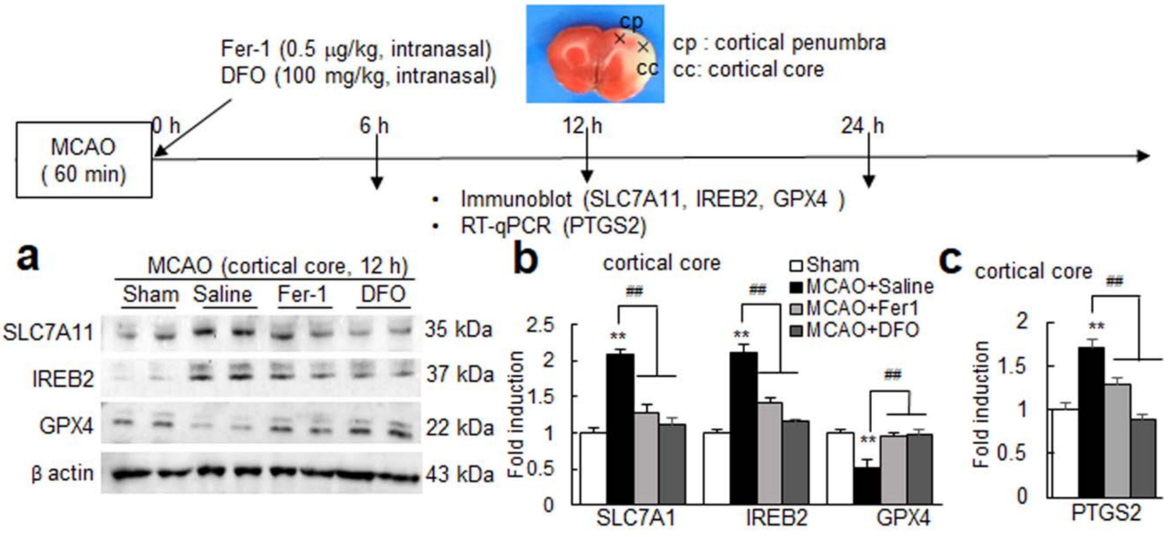

Supplementary Fig. 3. Ferroptosis in the post-ischemic brain (cortical cores)

Fer-1 (0.5 mg/kg) or DFO (100 mg/kg) were administered intranasally immediately after suture removal. (a-b) Levels of SLC7A11, IREB2, and GPX4 in cortical cores of ischemic hemispheres at 12 h post-MCAO were determined by immunoblotting. Representative images are presented in a and quantified results are presented as means ± SEMs (n=4) in b. (c) Levels of PTGS2 in cortical cores of ischemic hemispheres at 12 h post-MCAO were determined by RT-qPCR. Sham, sham-operated animals (n=4); MCAO+PBS, PBS-administered MCAO animals (n=4); MCAO+Fer-1, Fer-1-administered MCAO animals (n=4); MCAO+DFO, DFO-administered MCAO animals (n=4). **p < 0.01 vs. sham-operated group and $^{##}$p < 0.01 between indicated groups.

Supplementary Figure 4

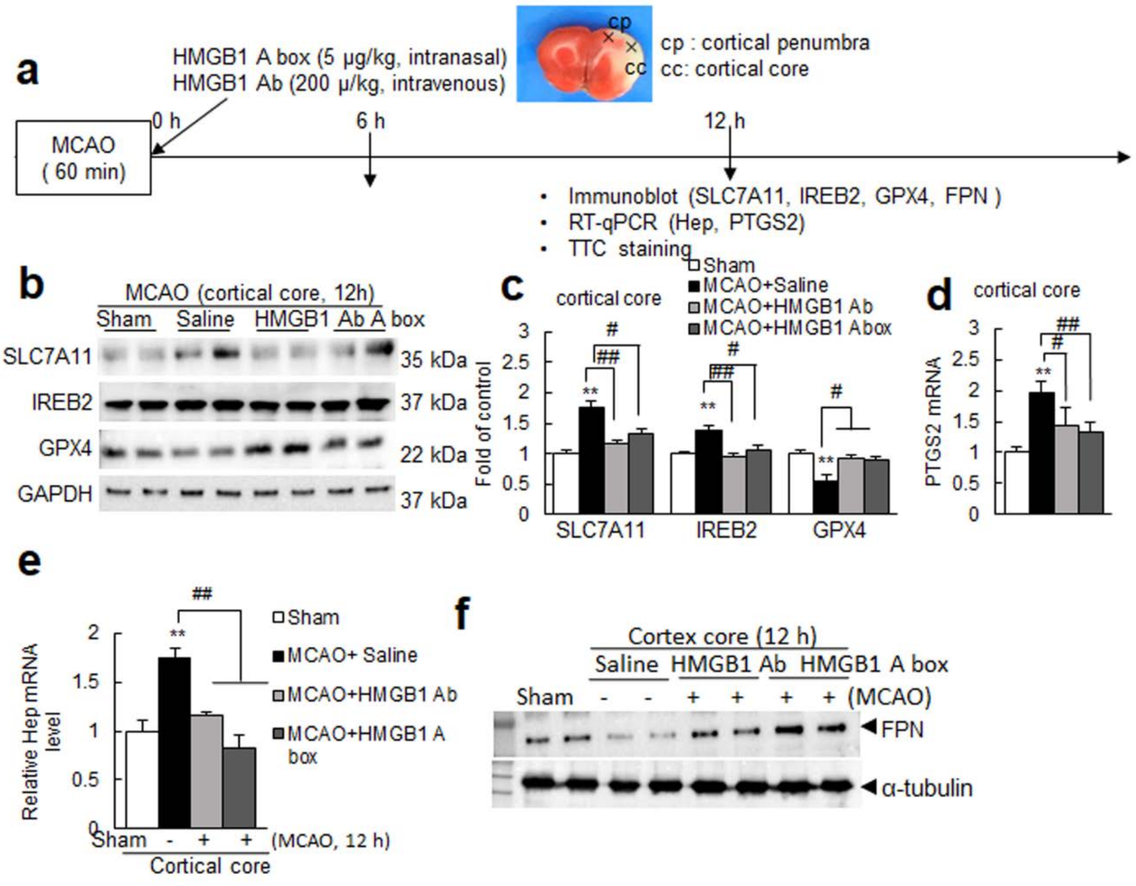

Supplementary Fig. 4. Blocking HMGB1 function with anti-HMGB1 antibody or HMGB1 A box suppressed ferroptosis in cortical cores in the post-ischemic brain

(a) HMGB1 A box (5 μg/kg) or anti-HMGB1 antibody (200 μg/kg) were administered intranasally and intravenously, respectively, immediately after suture removal. (b-c) Levels of SLC7A11, IREB2, and GPX4 in cortical cores of ischemic hemispheres at 12 h post-MCAO were determined by immunoblotting. Representative images are presented in b, and results are presented as means ± SEMs (n=4) in c. (d) Levels of PTGS2 in cortical cores of ischemic hemispheres at 12 h post-MCAO were determined by RT-qPCR. (e, f) Levels of Hep and FPN in the cortical cores of ischemic hemispheres at 12 h post-MCAO were determined by RT-qPCR and immunoblotting, respectively. Sham, sham-operated animals; MCAO+PBS, PBS-administered MCAO animals; MCAO+HMGB1 Ab, anti-HMGB1 Ab-administered MCAO animals; MCAO+HMGB1 A box, HMGB1 A box-administered MCAO animals. $^{**}p < 0.01$ vs. sham-operated group and $^{\#}p < 0.05$, $^{\#\#}p < 0.01$ between indicated groups.

Supplementary Figure 5

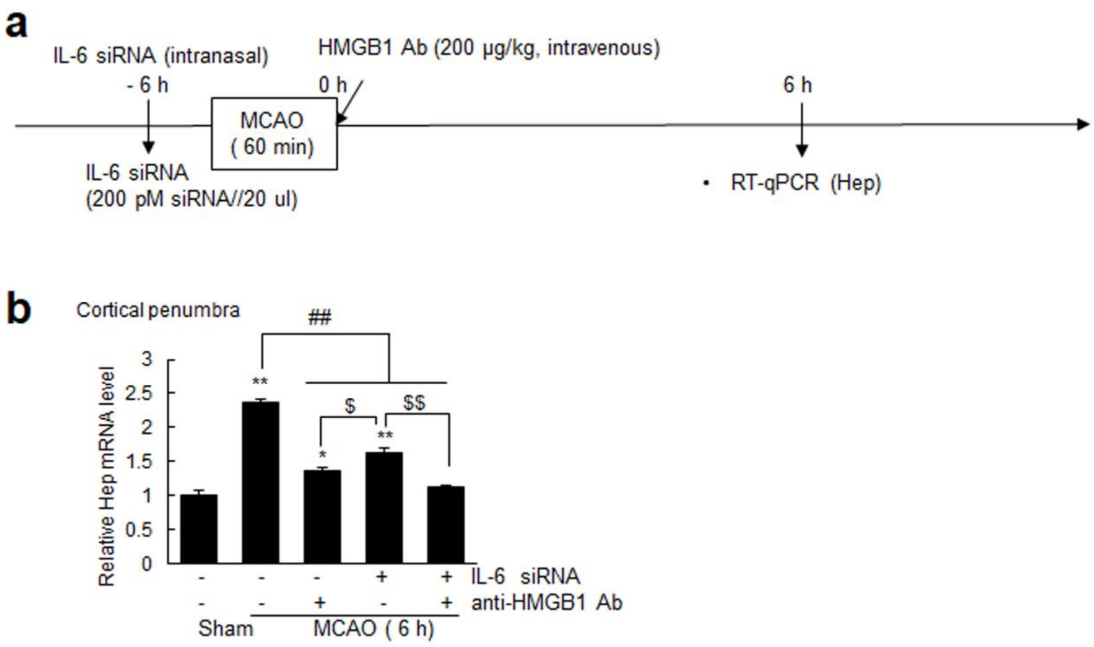

Supplementary Fig. 5. Contribution of HMGB1 to hepcidin upregulation in the post-ischemic brain in the absence of IL-6

(a) IL-6 siRNA (200 pM) dissolved in a 5% glucose solution was mixed with in vivo-jetPEI to an N/P ratio of 7, and a total of 20 µl of the siRNA-jetPEI complex was administered intranasally 6 h before MCAO. Anti-HMGB1 antibody (200 µg/kg) was administered intravenously immediately after suture removal. (b) Levels of hepcidin mRNA in the cortical penumbras of ischemic hemispheres at 6 h post-MCAO were determined by RT-qPCR. Sham, sham-operated animals; MCAO, saline-administered MCAO animals; MCAO+IL-6 siRNA, MCAO+IL-6 siRNA-administered MCAO animals; MCAO+HMGB1 Ab, anti-HMGB1 Ab-administered MCAO animals. *$p < 0.05$, **$p < 0.01$ vs. sham-operated group and $^{\#\#}p < 0.01$, $^{\$}p < 0.05$, $^{\$\$}p < 0.01$ between indicated groups.

Supplementary Figure 6

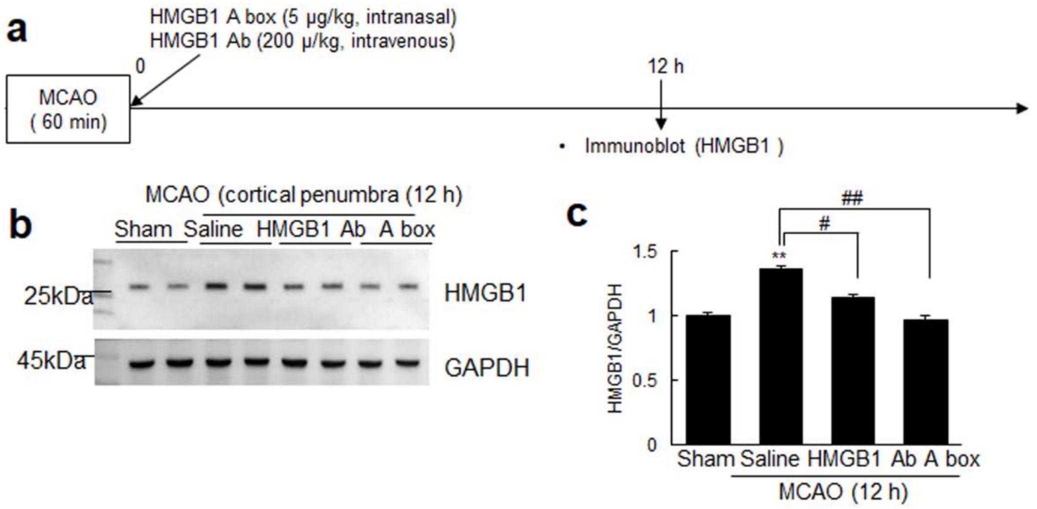

Supplementary Fig. 6. Administration of anti-HMGB1 antibody or HMGB1 A box suppressed HMGB1 induction in the post-ischemic brain

(a) HMGB1 A box (5 µg/kg) or anti-HMGB1 antibody (200 µg/kg) were administered intranasally and intravenously, respectively, immediately after suture removal. (b-c) Levels of HMGB1 in cortical penumbras of ischemic hemispheres at 12 h post-MCAO were determined by immunoblotting. Images are presented in b, and results are presented as means ± SEMs (n=4) in c. Sham, sham-operated animals; MCAO+Saline, Saline-administered MCAO animals; MCAO+HMGB1 Ab, anti-HMGB1 Ab-administered MCAO animals; MCAO+HMGB1 A box, HMGB1 A box-administered MCAO animals. $^{**}p < 0.01$ vs. sham-operated group and $^{\#}p < 0.05$, $^{\#\#}p < 0.01$ between indicated groups.

Supplementary Figure 7

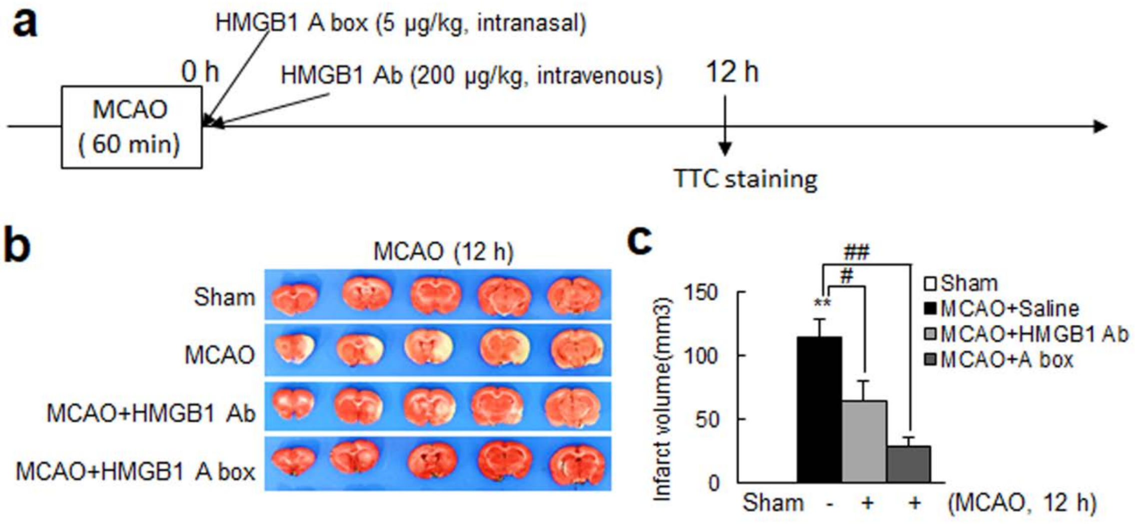

Supplementary Fig. 7. Blocking HMGB1 function with anti-HMGB1 antibody and HMGB1 A box suppresses infarct formation in the post-ischemic brain

(a) HMGB1 A box (5 μg/kg) and anti-HMGB1 antibody (200 μg/kg) was administered intranasally and intravenously, respectively, immediately after removing the suture. Coronal brain sections were prepared at 12 h post-MCAO and TTC staining was conducted (b) and mean infarct volumes were determined and are presented as means ± SEMs (c) (n=6). Sham, sham-operated animals; MCAO+PBS, PBS-administered MCAO animals; MCAO+HMGB1 Ab, anti-HMGB1 Ab-administered MCAO animals; MCAO+HMGB1 A box, HMGB1 A box-administered MCAO animals. **$p < 0.01$ vs. sham-operated group and $^{\#}p < 0.05$, $^{\#\#}p < 0.01$ between indicated groups.
